# Draft Genome Sequence of a Clinical *Enterococcus faecium* Sequence Type 18 Strain from South Africa

**DOI:** 10.1128/genomeA.01381-17

**Published:** 2017-11-30

**Authors:** Nontombi Marylucy Mbelle, Nontuthuko Excellent Maningi, Vhudzani Tshisevhe, Lesedi Modipane, Daniel Gyamfi Amoako, John Osei Sekyere

**Affiliations:** a Department of Medical Microbiology, Faculty of Health Sciences, University of Pretoria, Pretoria, South Africa; b National Health Laboratory Service, Pretoria, South Africa; c Biomedical Resource Unit, School of Laboratory Medicine and Medical Sciences, University of KwaZulu-Natal, Durban, South Africa; d Department of Pharmaceutics, Faculty of Pharmacy & Pharmaceutical Sciences, Kwame Nkrumah University of Science and Technology, Kumasi, Ghana

## Abstract

We report the first draft genome sequence of an *Enterococcus faecium* sequence type 18 (ST18) strain isolated from a tuberculosis patient in Africa. The genome is comprised of 3,202,539 bp, 501 contigs, 37.70% GC content, 3,202 protein-encoding genes, and 61 RNA genes. The resistome and virulome of this important pathogen are presented herein.

## GENOME ANNOUNCEMENT

*Enterococcus faecium* is a Gram-positive bacterium inhabiting the intestinal tract of humans and animals. This multidrug-resistant opportunistic pathogen is commonly associated with invasive nosocomial infections such as complicated urinary tract infection (UTI), bacteremia, and infective endocarditis (1). An *E. faecium* sequence type 18 (ST18) strain, ST-18:K073, was isolated from the urine of a 25-year-old female tuberculosis patient diagnosed with UTI in a hospital in South Africa (Pretoria) in 2013. Antimicrobial susceptibility testing (AST) with Vitek II showed it to be multidrug resistant.

ST-18:K073 was grown overnight anaerobically at 37°C in brain heart infusion (BHI) broth (Oxoid, UK) and was catalase negative but esculin hydrolysis and pyrrolidonyl arylamidase (PYR) positive. Genomic DNA of the *E. faecium* isolate was extracted and sheared to 200-bp libraries; 280-bp fragments were selected using 2% agarose gels and Pippen prep (Sage Science, Beverly, MA, USA). Individual libraries were pooled to 100 pM and sequenced on the Ion Proton (ThermoFisher, Waltham, MA, USA) at a coverage of 81.38×. The generated raw reads were *de novo* assembled using the SPAdes assembler (2).

The draft genome sequence has a total size of 3,202,539 bp with 37.70% GC content. The assembly contains 501 contigs with contig *N*_50_ and *L*_50_ values of 36,399 bp and 24 bp, respectively. Annotation with the prokaryotic genome annotation pipeline (PGAP) (3) found 3,528 protein-encoding genes and 326 (9.24%) hypothetical proteins. Genes coding for tRNA and rRNA were identiﬁed using tRNAscan (4) and RNAmmer (5), respectively. Approximately 61 tRNAs, 3 rRNAs, and 4 noncoding RNAs were identified. The CRISPRFinder (6) predicted 4 clustered regularly interspaced short palindromic repeat (CRISPR) arrays on nodes/contigs 30, 50, and 116 in the genome.

ST-18:K073 was confirmed as an *E. faecium* strain with the 16S rRNA-based species identification tool, SpeciesFinder (v1.2) (7). ST-18:K073 was similar to other *E. faecium* draft genomes, including strains K60-39 (GenBank accession no. CP023423), DO (CP003583), A_020709_82 (CP018128), and E745 (CP014529), all with ≥70% query cover and 99% identity.

GoSeqIt (8, 9) predicted six resistance genes, including two aminoglycosides [*aph(3′)-III* and *ant(6)-Ia*], two macrolide-lincosamide-streptogramin [*erm*(*B*)*, msr*(*C*)], and two tetracycline (*tetM* and* tetL*) resistance genes, which agreed with the AST profile, that is, resistant to gentamicin, streptomycin, erythromycin, clindamycin, tetracycline, ciprofloxacin, and moxifloxacin. As there were no ﬂuoroquinolone resistance genes identiﬁed, mutations in DNA gyrase (*gyrA*) and topoisomerases (*parC*), implicated in fluoroquinolone resistance (10), were found to mediate moxifloxacin and ciprofloxacin resistance. MLST v1.8 (11) showed *E. faecium* ST-18:K073 to be of sequence type 18 (ST18). The PlasmidFinder v1.3 (12) identified four plasmid replicon sequences (rep2, rep14, repUS12, and repUS15). The VirulenceFinder database (8) identified two putative virulence factors, namely, collagen binding protein (acm) and cell wall adhesion (*efaAfm*) in the ST-18:K073 genome, contributing to the strain’s ability to adhere to and escape from their host. This whole-genome sequence provides insights into the resistance, virulence, and pathogenicity of *Enterococcus* spp.

### Accession number(s).

This draft genome sequence has been submitted to NCBI/GenBank under the accession no. NXIX00000000. The version described in this paper is version NXIX01000000.
